# Plantar forefoot pressures in psoriatic arthritis-related dactylitis: an exploratory study

**DOI:** 10.1007/s10067-016-3304-z

**Published:** 2016-05-26

**Authors:** Richard A. Wilkins, Heidi J. Siddle, Anthony C. Redmond, Philip S. Helliwell

**Affiliations:** 1 Section of Clinical Biomechanics and Physical Medicine, Leeds Institute of Rheumatic and Musculoskeletal Medicine, 2nd Floor, Chapel Allerton Hospital, Chapeltown Road, Leeds, LS7 4SA UK; 2 Foot Health Department, Leeds Teaching Hospitals NHS Trust, Lower Ground Floor, Chancellor Wing, St James’s University Hospital, Beckett Street, Leeds, LS9 7TF UK; 3 Rheumatology Department, Bradford Teaching Hospitals NHS foundation Trust, St Luke’s Hospital, Little Horton Lane, Bradford, BD5 0NA UK

**Keywords:** Dactylitis, Plantar pressure measurement, Psoriatic arthritis, Toe

## Abstract

Dactylitis is a common feature of psoriatic arthritis (PsA); local physical trauma has been identified as a possible contributing factor. The aim of this study was to explore differences in forefoot plantar pressures in patients with PsA with and without dactylitis and compare to healthy controls. Thirty-six participants were recruited into three groups: group A PsA plus a history of dactylitis; group B PsA, no dactylitis; group C control participants. Forefoot plantar pressures were measured barefoot and in-shoe at the left second and fourth toes and corresponding metatarsophalangeal joints. Temporal and spatial parameters were measured and data from the foot impact scale for rheumatoid arthritis (FIS-RA), EQ5D and health assessment questionnaire (HAQ) were collected. Pressure time integral peak plantar pressure, and contact time barefoot and in-shoe were not significantly different between groups. Temporal and spatial parameters reported no significant differences between groups. ANOVA analysis and subsequent post hoc testing using Games-Howell test yielded significance in FIS-RA scores between both PsA groups versus controls, A *p* ≤ 0.0001 and PsA group B *p* < 0.0001 in the FIS-RA impairment and footwear domain, PsA group A *p* < 0.03 and PsA group B *p* ≤ 0.05 in the FIS-RA activity and participation domain compared to controls. This is the first exploratory study to investigate forefoot plantar pressures in patients with and without historical dactylitis in PsA. FIS-RA scores indicate PsA patients have significant limitations compared to controls, although a history of dactylitis does not appear to worsen patient reported outcomes.

## Introduction

Levels of peripheral joint damage are lower in psoriatic arthritis (PsA) compared to rheumatoid arthritis (RA), although foot impairment and disability are reported in two thirds of patients particularly at the forefoot [1]. Patients report a reduction in quality of life, with limitations in function and disability described as equivalent to that of RA. Despite lower levels of peripheral joint damage however the effect on patients reported pain and disability are significant [2, 3]. Dactylitis is one of the most common features of PsA occurring in around 40 % of cases. It is classed as a hallmark feature of the disease and forms part of the classification criteria for PsA [4]. Dactylitis, also referred to as sausage digit presents clinically as an acute painful inflammation of the digit which in the chronic phase can remain swollen following the subsidence of acute inflammation.

Magnetic resonance imaging (MRI) studies have identified dactylitis as a polyarticular disease process, with multiple pathologies and varying levels of severity. Bone oedema and flexor tenosynovitis have been observed and, to a lesser degree, extensor tenosynovitis. Furthermore, synovitis and soft tissue oedema occur in tender and non-tender dactylitis [5]. Trauma has been suggested as a potential trigger for PsA and direct physical injury may influence peripheral manifestations such as dactylitis and enthesitis. Furthermore, high levels of stress at entheses are suggested as a biomechanical trigger to enthesitis and the other manifestations of the disease [6].

It has been hypothesised that dactylitis in the hand may be caused by mechanical trauma to distal phalangeal and metacarpal joints. This is thought to result in an inflammatory response at the digit, known as the ‘deep Koebner’ phenomenon [4, 7, 8]. Toe dactylitis is more common than finger dactylitis which may support the mechanical pathogenesis hypothesis given the load bearing function of the toes, but there are no investigations to support a mechanical trigger [7, 8]. It has also been suggested that psoriatic nail disease may be linked to micro trauma occurring within the nail bed [9].

Pain at the forefoot has been reported in RA to reduce the ambulatory performance in the presence of foot deformity and pain. Localised foot pain may lead to altered changes in temporal and spatial parameters of gait leading to altered gait patterns and functional adaption [10, 11]. During normal walking, the toes function to increase the total weight-bearing area of the forefoot and disperse the mechanical load from the metatarsal-phalangeal (MTP) joints [12]. At the propulsive phase of gait where forefoot forces are highest, muscle activation occurs to facilitate propulsion. Force generated at the forefoot and musculature is increased, which in inflammatory diseases such as PsA maybe abnormal leading to changes in plantar pressures [12, 13]. Although the role of plantar MTP joint pressure distributions have been investigated in PsA, no study has investigated toe dactylitis [14]. In other systemic conditions, such as RA and diabetes, increases in pressure and the time over which pressure occurs have been linked to mechanical tissue damage and ulceration [10, 15, 16]. To the authors’ knowledge, no study has investigated the effect of toe dactylitis.

It is therefore the aim of this exploratory study to investigate variations in plantar pressures at the most commonly reported sites of dactylitis and their corresponding MTP joints in patients with a history of dactylitis when compared to controls (PsA, no history of dactylitis) and normal participants.

## Method

Ethical approval was obtained from the NRES Committee Yorkshire and Humber—Leeds East. Written informed consent was obtained from all participants.

The study was cross-sectional in design with 36 participants recruited as a convenience sample of consecutive patients identified by two consultant rheumatologists. Twelve participants with PsA and a previous history of dactylitis/chronic dactylitis (group A) and 12 participants with PsA but no previous history of dactylitis (group B) were recruited from the rheumatology outpatient department, Chapel Allerton Hospital, Leeds Teaching Hospitals NHS Trust. A control group of 12 heathy participants were also recruited (group C). These control participants did not report any musculoskeletal or rheumatological disease and had no current or medical history of foot and ankle pain. Control participants were age (+/− 2 years) and gender matched. Disease duration (years) was recorded for all patients with PsA. The sites of historical dactylitis (digit number, left/right) were recorded for all PsA patients in group A.

The EQ-5D was used to capture participant health status and self-rated health. The health assessment questionnaire (HAQ) was also used and provided a measure of the patient’s health, functional status, symptoms, and quality of life from the participants’ own perspective. Both EQ-5D and HAQ have been reported in PsA previously (ref). The foot impact scale for rheumatoid arthritis (FIS-RA) was used to measure the impact of foot pathology on impairment and footwear (FIS-RA_IF_), and activity limitation and participation restriction (FIS-RA_AP_) [17]. Although the FIS-RA is not specific to PsA it has been used previously [2].

Temporal and spatial parameters of walking were collected using the GAITRite system, a 10-metre instrumented walkway. Barefoot plantar pressure measurement and dynamic foot/shoe interface pressures were collected using the novel eMED SF and Pedar in-shoe systems (Novel GmbH, Munich, Germany), respectively. Peak plantar pressure (PPP) (kPa), contact area (CA) (cm^2^) and pressure time integral (PTI) (kPa/s) were analysed at the most common (fourth toe) and second most common (second toe) sites of dactylitis and corresponding metatarsophalangeal (MTP) joints. Three representative steps were collected for barefoot pressures using a common two-step start protocol [18, 19]. Dynamic in-shoe pressure was collected by inserting a flexible pressure measuring insole into the participant’s footwear. The participant undertook two straight line walks of an 8-m walkway generating 20 representative steps [19].

### Data analysis

**Fig. 1:**
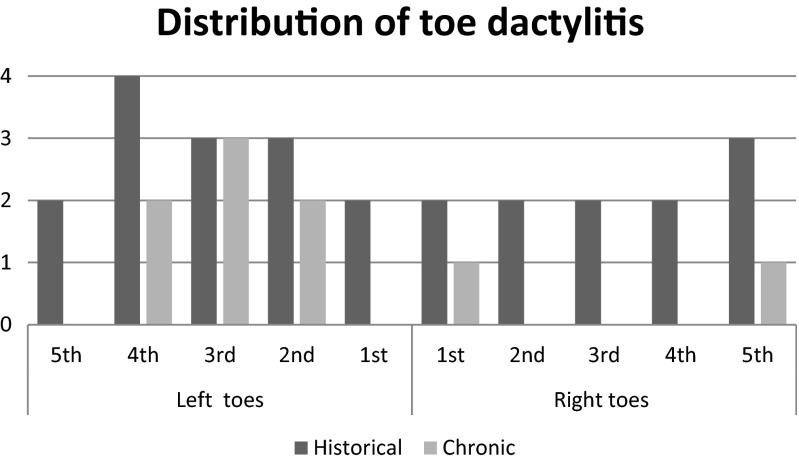
Distribution of current (chronic) and historical dactylitis in both feet

A sample size of 36 was used (12 participants per group), based on a rationale including feasibility, precision about the mean and variance and regulatory considerations for sample size calculations [20]. The most symptomatic foot (left) in group A (PsA dactylitis) was selected for analysis (Fig. 1). IBM Statistical package for Social Sciences (SPSS) Version 19 for Windows 7 was used to analyse data. Descriptive statistics were used to report patient demographics, between-group spatial and temporal parameters (gait velocity (m/s), cadence (steps/min) and period of double support (%)). Between-group differences were explored using a one-way analysis of variance (ANOVA) for PPP (kPa), PTI (kPa/s) and the FIS-RA. A subsequent Games-Howell post hoc test explored differences between paired combinations of the three groups (A and B, B and C, C and A). A *p* value of ≤0.05 was chosen to detect the probability.

## Results

Thirty-six participants were analysed for this exploratory study. Patient demographics, patient-reported outcome measures and temporal and spatial data are presented descriptively in Table 1.

**Table 1:** Demographics, disease variables and patient reported outcome measures

	Group		
	PsA dactylitis (group A)	PsA no history of dactylitis (group B)	Normal (group C)
Demographics (SD) (range)			
Gender M:F	11:1	4:8	7:5
Age, mean	36.7 (21.5) (25–58)	45.3 (16.2) (20–81)	39.7 (8.8) (29–58)
Disease duration (y)	4.6 (6.7) (1–25)	4.6 (5.6) (1–20)	0 (0)
Weight (kg)	90.9 (21.5) (54–133)	81.3 (29.8) (51–162)	71.8 (15) (48–98)
Height (m)	1.8 (0.1) (1.6–1.9)	1.7 (0.1) (1.5–1.9)	1.7 (0.1) (1.8–1.9)
Medication (n)			
DMARDs	10	11	0
Biologics	7	6	0
FIS-RA (SD) (range)			
FIS-RA (SD) (range)			
FIS-RA_IF_	7.16 (5.78) (0–15)	6.83 (4.19) (0–12)	0.41 (0.79) (0–2)
95 % CI lower bound	3.49	4.16	−0.08
95 % CI upper bound	10.84	9.49	0.92
FIS-RA_AP_	8.75 (10.22) (0–25)	5.75 (7.30) (0–22)	0.16 (0.38) (0–1)
95 % CI lower bound	2.25	1.11	−0.08
95 % CI upper bound	15.24	10.38	0.41
EQ-5D			
Mobility	1.42 (0.51)	1.50 (0.52)	1.0 (0)
Self-care	1.25 (0.45)	1.08 (0.28)	1.0 (0)
Activity	1.50 (0.67)	1.50 (0.52)	1.08 (0.28)
Pain	1.67 (0.49)	1.67 (0.49)	1.17 (0.38)
Anxiety	1.25 (0.45)	1.33 (0.49)	1.00 (0)
VAS	66.67 (23.17)	78.33 (14.63)	89.78 (9.9)
HAQ	0.45 (0.84)	0.30 (0.41)	0.03 (0.10)

Descriptive of HAQ and EQ-5D are reported in Table 1. There were no significant differences between groups. In the FIS-RA a mean FIS-RA_IF_ score of 7.16 in group A and 6.83 in group B compared to 0.41 in the control group, and a mean FIS-RA_AP_ score of 8.75 in group A and 5.75 in group B compared to 0.16 in the control group; ANOVA analysis and subsequent post-hoc testing identified statistically significance differences between groups in FIS-RA scores (Table 2). In both domains of the FIS-RA, there was a significant difference between both PsA groups compared to the control group; PsA group A *p* = 0.0001 and PsA group B *p* = 0.0001 in the FIS-RA_IF_ domain, PsA group A *p* = 0.03 and PsA group B *p* = 0.05 in the FIS-RA_AP_ domain.

**Table 2:** Post hoc test Games-Howell FIS-RA subscales

	Groups					
	PsA dactylitis (group A) (1)		PsA no history of dactylitis (group B) (1)		Normal (group C) (1)	
	PsA no history of dactylitis (group B) (2)	Normal (group C) (2)	PsA dactylitis (group A) (2)	Normal (group C) (2)	PsA dactylitis (group A) (2)	PsA no history of dactylitis (group B) (2)
FIS-RA_IF_						
Mean difference (1and 2)	0.33	6.75	−0.33	1.23	1.68	1.23
Sig.	0.986	0.00	0.986	0.00	0.00	0.00
95 % CI lower bound	−4.88	2.28	−5.54	3.11	−11.27	−9.71
95 % CI upper bound	5.54	11.27	4.88	9.71	−2.22	−3.11
FIS-RA_AP_						
Mean difference (1 and 2)	3.00	8.58	−3.00	5.58	−8.58	−5.58
Sig.	0.691	0.03	0.69	0.05	0.03	0.05
95 % CI lower bound	−6.179	0.60	−12.17	−0.11	−16.55	−11.27
95 % CI upper bound	12.17	16.55	6.17	11.27	−0.60	0.11

Descriptive statistics of mean plantar pressure variables (PPP, PTI, CA) are reported in Table 3. ANOVA analysis of measurements barefoot and in-shoe (Table 4) indicated no significant differences between groups. No significant differences were reported in spatial and temporal parameters of gait between groups (Table 1).

**Table 3:** Gait and plantar pressure descriptive statistics

Temporal and spatial (SD) (range)			
Velocity (m/s)	1.23 (0.17) (0.59–1.44)	1.23 (0.24) (0.59–1.44)	1.47 (0.14) (1.28–1.75)
Cadence	116.8 (7.34) (103–125)	120.03 (9.26) (105–138)	120.26 (5.57) (110–128)
Double support (% of gait cycle left)	22.56 (3.17) (17–28)	23.67 (4.40) (19–33)	19.55 (2.31) (15–22)
Double support (% of gait cycle right)	22.61 (3.19) (18–28)	23.40 (4.42) (20–33)	19.27 (2.36) (15–22)
PP/PTI/CA Emed-SF (SD) (range)			
PP left 2nd toe	296.6 (238) (62–952)	197.3 (97.3) (0–317)	245.6 (128.8) (68–455)
PP left 4th toe	88.2 (55.8) (15–232)	81.4 (90.2) (0–292)	122.7 (82.9) (10–272)
PP left 2nd MTP joint	555.3 (260.3) (110–1035)	601.8 (242.8) (268–1047)	633.6 (305.5) (353–1235)
PP left 4th MTP joint	372.8 (203.8) (75–753)	330.4 (128.7) (208–698)	314.5 (104) (170–492)
PTI left 2nd toe	64.3 (54.2) (18–211)	38.9 (21.1) (0–64)	51.2 (37.8) (13–158)
PTI left 4th toe	19.5 (13.2) (1–50)	19.3 (24.9) (0–83)	28.5 (20.8) (1–64)
PTI left 2nd MTP joint	162.4 (64.4) (17–259)	188 (88.3) (66–330)	167.3 (62.3) (92–288)
PTI left 4th MTP joint	135.3 (74.9) (24–267)	110.4 (38.7) (76–212)	100.8 (33.9) (58–181)
Contact area left 2nd toe	3.78 (.50) (0–2)	3.39 (1.34)	3.70 (.93)
Contact area left 4th toe	2.52 (.88)	1.96 (1.52)	2.68 (.92)
Contact area left 2nd MTP joint	9.82 (1.43)	9.90 (1.81)	9.70 (1.14)
Contact area left 4th MTP joint	9.11 (1.86)	8.89 (1.49)	8.87 (1.30)
PP/ PTI/CA PEDAR (SD) (range)			
PP left lesser toes	117.9 (45.1) (54–194)	110 (42.2) (24–162)	108.7 (43.6) (64–228)
PP Left MTP joints	301.9 (68.2) (165–454)	271.4 (100.8) (164–482)	300.7 (71.8) (215–455)
PTI left lesser toes	301.9 (68.2) (165–454)	271.4 (100.8) (164–482)	300.7 (71.8) (215–455)
PTI left MTP joints	84.12 (35.2) (54–167)	74.5 (30.9) (37–128)	68.32 (14.6) (45–96)
CA left lesser toes	8.1 (1.1) (3.7)	6.6 (2.5) (10.3)	7.4 (0.9) (2.9)
CA left MTP joints	18.4 (2.9) (11.5)	15.5 (5.3) (21.3)	17.4 (3.3) (10.6)

**Table 4:** ANOVA one-way analysis of emed-SF barefoot and PEDAR in-shoe data contact area (CA), peak plantar pressures (PPP) and pressure time integral (PTI)

		Mean square	Sig.
PPP left 2nd toe	Between groups	5035.533	0.43
	Within groups	5961.038	
PPP left 4th toe	Between groups	15499.007	0.48
	Within groups	21111.535	
PPP left 2nd MTP joint	Between groups	17317.560	0.78
	Within groups	69591.536	
PPP left 4th MTP joint	Between groups	37784.462	0.27
	Within groups	27993.069	
PTI left 2nd toe	Between groups	37784.462	0.27
	Within groups	27993.069	
PTI left 4th toe	Between groups	37784.462	0.27
	Within groups	27993.069	
PTI left 2nd MTP joint	Between groups	37784.462	0.27
	Within groups	27993.069	
PTI left 4th MTP joint	Between groups	37784.462	0.27
	Within groups	27993.069	
CA left 2nd toe	Between groups	0.936	0.40
	Within groups	0.986	
CA left 4th toe	Between groups	1.719	0.28
	Within groups	1.315	
CA left 2nd MTP joint	Between groups	0.123	0.85
	Within groups	2.228	
CA left 4th MTP joint	Between groups	0.225	0.91
	Within groups	2.475	
PEDAR PPP left toes	Between groups	291.323	0.85
	Within groups	1908.989	
PEDAR PPP left MTP joints	Between groups	3369.648	0.60
	Within groups	6543.641	
PEDAR PTI left toes	Between groups	23.181	0.81
	Within groups	112.885	
PEDAR PTI left MTP joints	Between groups	759.532	0.39
	Within groups	797.616	
PEDAR CA left MTP toes	Between groups	6.483	0.11
	Within groups	2.697	
PEDAR CA left MTP joints	Between groups	24.984	0.22
	Within groups	15.485	

## Discussion

This is the first study to explore the mechanical factors that may contribute to toe dactylitis in patients with PsA and to examine the impact of dactylitis on foot pain and disability. Patients with PsA, with and without dactylitis, reported worse impairment, footwear, activity limitation and participant restriction when compared to a control group of normal participants. Analysis of peak plantar pressure, pressure time integral and contact area at the 2nd and 4th toes, and 2nd and 4th MTP joints of the left foot found no significant differences between groups. Descriptive analysis of temporal and spatial parameters of gait identified no differences between any of the three groups (A, B, C). Having a history of dactylitis did not have a significant effect on plantar pressure measurement and patient-reported foot impact in patients with PsA.

This study in PsA has provided an insight in to the mechanical factors that may be associated with toe dactylitis and aided the formation of a hypothesis for a future study. Although differences in PsA PPP, PTI and contact area were not identified, the results indicate that pressure may not be as relevant to the cause of trauma as previously hypothesised. In other diseases, such as RA, inflammatory changes at the forefoot leads to altered joint mechanics and increased plantar pressures [10]. This was not the finding of this study and supports the research carried out by Turner et al. who reported plantar pressures at the MTP joint did not correlate to joint damage and pain in patients with PsA [14]. Active or historical dactylitis did not correlate with changes in plantar pressures. Investigating the effect of dorsal and plantar shear force on the forefoot structures in the future may provide more insight into mechanical properties of soft tissue.

### Limitations

The authors acknowledge several limitations to this study. Whilst a sample size of 12 per group is accepted in an exploratory study the inability to adequately power may increase the risk of error. A power calculation using the new data indicates that 60 participants per group would be needed to demonstrate a significant difference between groups if these results were found. Capturing details of disease activity may have provided more insight into the condition and the patient disease status at the point of data collection such as active systemic inflammation which may affect the patient’s ability to weight bear through the forefoot. Including both chronic and historical dactylitis may have affected results. The inclusion of historical dactylitis in group A may have negatively impacted on the results and lessened the impact that current dactylitis may have had on gait parameter and altered function. Details of foot deformity, foot posture and footwear characteristics may also provide insight into plantar pressure data reported and contributing factors such as foot type, poor fitting footwear or poor mechanical properties.

## Conclusion

In conclusion, the main finding of this exploratory study is that patient-reported functional limitation and disability in PsA is significant, regardless of whether or not there is a history of dactylitis. Further research is necessary in larger patient numbers using more sophisticated measures, and capturing variables of foot type, foot deformity and footwear in conjunction with disease activity scores to determine whether variations in plantar pressures contribute to the manifestation of dactylitis in the toes of patients with PsA.
